# Sydnone C-4 heteroarylation with an indolizine ring via Chichibabin indolizine synthesis

**DOI:** 10.3762/bjoc.12.245

**Published:** 2016-11-23

**Authors:** Florin Albota, Mino R Caira, Constantin Draghici, Florea Dumitrascu, Denisa E Dumitrescu

**Affiliations:** 1 Center of Organic Chemistry ‘‘C. D. Nenitzescu’’, Roumanian Academy, Spl Independentei 202B, 060023 Bucharest, Romania; 2 Department of Chemistry, University of Cape Town, Rondebosch 7701, South Africa; 3 Faculty of Pharmacy, University Ovidius, Aleea Universitatii 1, Constantza, Romania

**Keywords:** biheteroaryl, Chichibabin synthesis, indolizine, pyridinium *N*-ylide, sydnone

## Abstract

The synthesis of sydnones heteroarylated at C-4 with an indolizine was achieved by Chichibabin (Tschitschibabin) indolizine synthesis starting from the corresponding sydnone-*N*-pyridinium bromides. The latter compounds were also transformed to sydnone-indolizines connected through a keto group at the C-4 position by refluxing them in 1,2-epoxybutane with an activated alkyne. The structures of the new compounds were assigned by FTIR, NMR spectroscopy and X-ray analysis.

## Introduction

In recent decades, interest in the syntheses of biheteroaryls has been focused on the creation of new hetaryl–hetaryl C(sp^2^)–C(sp^2^) bonds, in particular through cross-coupling reactions. These reactions are catalyzed by palladium or other metals and represent a versatile tool for obtaining hybrid heterocycles with properties that are useful for the development of new advanced materials and for medicinal purposes [1–4].

Recently, we obtained, by 1,3-dipolar cycloaddition reactions of sydnone-ylides as bis(1,3-dipoles) with activated acetylenes, the hybrid structures **1** containing a pyrroloazine (pyrroloisoquinoline or pyrrolophthalazine) connected to the sydnone ring through a keto group [5]. As a result, it was of interest to prepare analogous compounds containing a pyrroloazine directly attached to the C-4 position of a sydnone ring. From a survey of the literature data [6–15] and based on our previous findings [16–19] it was concluded that the heterocycle in the pyrroloazine series most suitable for this study would be indolizine (**2**).

Indolizine or pyrrolo[1,2-*a*]pyridine (**2**) and its derivatives exhibit remarkable biological activities and optical properties. Indolizine is a privileged structure for the design of new drugs and its framework is present in many natural compounds. As a consequence, the synthesis, chemical, physicochemical and biological properties of indolizines have been the subject of various reviews [6–15].

Sydnones **3** are mesoionic heterocyclic compounds with a 1,2,3-oxadiazole skeleton and their chemistry and biological properties are well represented and documented in the literature [20–26]. The most interesting chemical reactions of sydnones are their electrophilic substitution at C-4 and their participation in 1,3-dipolar cycloaddition reactions with olefinic and acetylenic dipolarophiles to form pyrazoles and functional transformation at C-4 [20–26].

**Figure 1:**
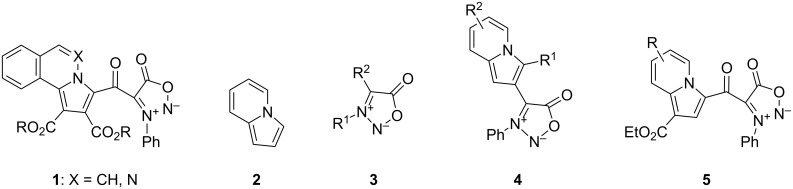
Sydnone-pyrroloazines hybrids **1**, indolizine (**2**), sydnone **3**, indolizines attached directly to C-4 of a sydnone **4**, indolizines attached via a keto group to C-4 of a sydnone **5**.

Herein we present the synthesis of indolizines **4** (Figure 1) with the indolizine directly attached to C-4 of a sydnone ring by the indolizine Chichibabin approach. In addition, the new indolizine structures **5**, connected through a keto group to a sydnone ring, were prepared by 1,3-dipolar cycloaddition reaction starting from the same pyridinium salts used in the indolizine Chichibabin synthesis.

## Results and Discussion

In order to obtain the compounds **4** containing an indolizine ring attached directly to the C-4 carbon atom of a sydnone ring (i.e., indolizination of sydnones at C-4), the Chichibabin indolizine synthesis was employed. The Chichibabin reaction was discovered ninety years ago [27] and consists in the cyclization of pyridinium salts that require the presence of a CH_2_R group (e.g., R = H, Alk, Ar, CN, CO_2_Et, SO_2_Ar) at the position adjacent to the nitrogen atom. Despite this limitation, the method remains one of the most efficient syntheses of indolizines [28–31] owing to the simple working procedure and the large number of 2-substituted pyridines that are commercially available. An overview of Chichibabin indolizine syntheses has been included in the reviews dedicated to indolizine syntheses and their applications in medicine and advanced materials [6–15].

### Synthesis of pyridinium bromides **8**

**Scheme 1:**
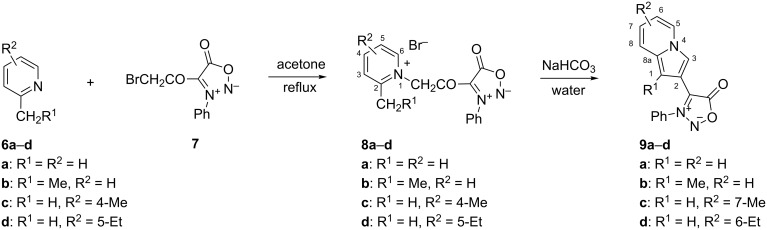
Synthesis of pyridinium bromides **8** and sydnone-indolizine hybrids **9** through the Chichibabin reaction.

The pyridinium bromides **8** were the starting materials for the synthesis of indolizines attached either directly or through a keto group to the sydnone ring. These salts were obtained in good yields by heating pyridines **6a–d** with 4-(bromoacetyl)-3-phenylsydnone (**7**, Scheme 1) in refluxing acetone [5]. The obtained pyridinium salts **8** were used in the next step without further purification.

The structures of the pyridinium bromides **8a**–**d** were assigned by ^1^H NMR, ^13^C NMR and IR spectroscopy. The chemical shifts for the protons of the pyridine ring match the proposed structures **8**. In the ^1^H NMR spectra of the pyridinium bromides recorded in DMSO-*d*_6_ the protons of the methylene groups appear as singlets with chemical shifts in the range of 5.96–6.06 ppm. The pyridine ring protons are strongly deshielded and were assigned based on their multiplicity and by comparison with literature data for compounds with similar structures. Thus, the chemical shifts of H-4 (8.44–8.60 ppm) and H-6 (8.72–8.93 ppm) are more deshielded due to their γ and α positions, respectively, to N-1 in comparison with H-3 (7.91–8.10 ppm) and H-5 (7.82–8.04), which are both in β position relative to N-1.

In the ^13^C NMR spectrum pyridinium bromides **8** representative chemical shifts are found for the methylene group (62.7–63.4 ppm), C-4 from the sydnone ring (106.2–106.3 ppm) and the two carbonyl groups. Here, the chemical shifts for the endocyclic C=O of the sydnone ring lie in the range of 165.7–165.8 ppm whereas for the exocyclic C=O the chemical shifts are at ~176.5 ppm. The chemical shifts for C-2, C-4 and C-6 (145.4–160.4 ppm) in the α and γ positions of the pyridine ring are easily identified as they are deshielded in comparison with C-3 and C-5 which are in the β position (125.4–129.7 ppm). An exception to the rule is the chemical shift for C-5 of compound **8d**, which has a high value of 141.3 ppm due to the influence of the ethyl substituent attached to C-5. In the ATR-IR spectra of compounds **8** the functional group absorption frequencies are representative for the CO groups attached to C-4 of the sydnone ring (range 1678–1683 cm^−1^) and the absorption bands associated with CO groups from the endocyclic sydnone ring (range 1771–1779 cm^−1^).

### Indolizination of sydnones at C-4 by the Chichibabin reaction

Generally, the formation of single bonds between two aromatic or heteroaromatic rings involves palladium-catalyzed cross-coupling reactions and cyclization of functional groups attached to an aromatic and heteroaromatic ring. Thus, arylation and heteroarylation at the C-4 position of sydnones has been performed by palladium-catalyzed cross-coupling reactions [32–36] starting from 4-unsubstituted sydnones, 4-halosydnones or by chemical transformation of the substituents attached to C-4 [32–36].

Herein, using the Chichibabin indolizine reaction one of the simplest and most efficient methods for indolizine synthesis, the “indolizination” at the C-4 position of sydnones was achieved using a procedure involving quaternization of pyridines **6** with sydnone derivative **7** to form pyridinium bromides **8**, followed by their intramolecular cyclization in the presence of a base. In order to improve the yield, the transformation of pyridinium bromides **8** into the sydnone-indolizine biheteroaryls **9** (Scheme 1) was optimized by some variations in the Chichibabin indolizine synthesis for the representative salt **8a**. Thus, the treatment of the quaternary salts **8** dissolved in hot water with sodium bicarbonate followed by solvent extraction gave the sydnone-indolizines **9** in yields of over 50%. If the dissolution of the quaternary salts was incomplete, a small quantity of ethanol could be added. Alternatively, replacement of sodium bicarbonate with 1,8-diazabicyclo[5.4.0]undec-7-ene (DBU) and the use of acetonitrile as solvent gave, in the case of the representative pyridinium bromide **8a**, compound **9a** in 53% yield. Similarly, **9a** could be prepared in 54% yield by heating pyridine **6a** under reflux with sydnone derivative **7** for four hours followed by the addition of DBU for cyclization. Although all three procedures for the cyclization of bromides **8** finally gave good results, the method involving water as reaction medium and sodium bicarbonate as cyclization reagent was preferred because it used non-toxic components. The cyclization of pyridinium bromides **8** to biheteroaryls **9** allows the isolation of compounds with substituents in both rings and has the advantage over cross-coupling reactions insofar as it overcomes side reactions and uses inexpensive materials.

**Figure 2:**
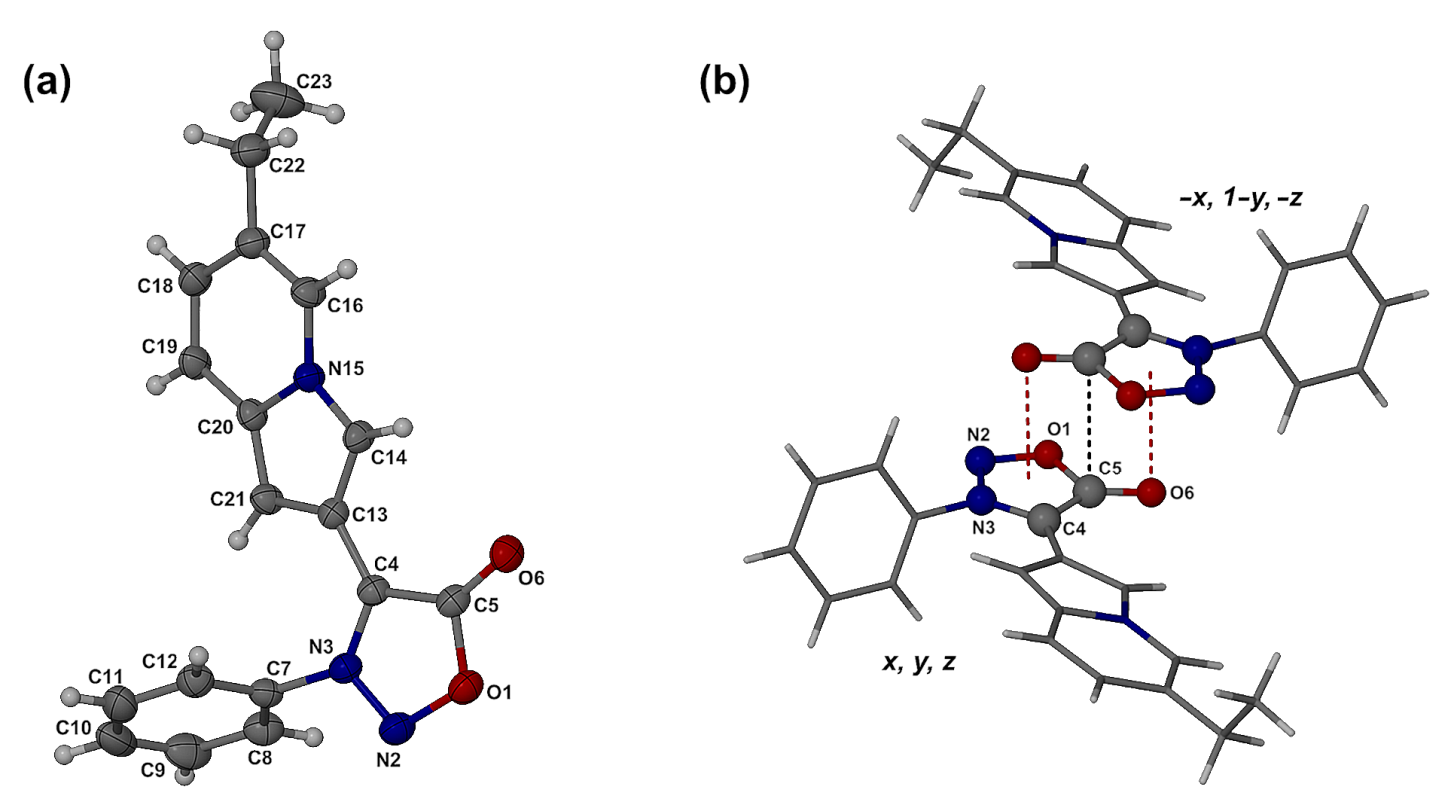
The molecular structure of **9d** with thermal ellipsoids drawn at the 50% probability level. (a) Inversion-related molecules of **9d** showing the antiparallel alignment of the dipolar sydnone rings and short contacts C5···C5^i^ (i = *−x, 1−y, −z*) and O6···ring centroid, represented by dashed lines. (b) Bond distances (Å) in the sydnone moiety are: O1–N2 1.382(1), N2–N3 1.322(2), N3–C4 1.349(2), C4–C5 1.414(2), C5–O1 1.402(2), and C5–O6 1.222(2).

The structures of the new sydnone-indolizine hybrids produced by the Chichibabin synthesis were confirmed by elemental analysis, NMR and IR spectroscopy, with further supporting X-ray structural elucidation of the representative compound **9d**, which displays an interesting packing feature described below. The molecular structure and numbering scheme for **9d** are shown in Figure 2a. Principal torsion angles that describe the molecular conformation include C5–C4–C13–C14, −18.2(2)° and C4–N3–C7–C8, 111.2(2)°, defining the orientations of the indolizine and phenyl rings relative to the sydnone ring. Bond distances in the latter ring (see caption of Figure 2) compare favorably with previously published values in the Cambridge Structural Database (CSD) [37]. The five-membered ring is planar, with a maximum deviation from the least-squares plane of 0.011(1) Å for C5, while atom O6 deviates from the plane by 0.022(1) Å. Full listings of geometrical parameters are available in the CIF file (Supporting Information File 1).

A search of the CSD revealed 104 crystal structures that contain the sydnone moiety. Of these, eleven were found to display the same anti-parallel sydnone–sydnone motif as is occurring in **9d** (Figure 2b), reflecting the dipolar alignment of these moieties consistent with the positive charge centered on the ring and the negative charge located on the exocyclic oxygen atom. This robust supramolecular motif is characterized by an abnormally short C5···C5′ distance for the entries listed in the CSD, namely 3.088–3.338 Å. In the crystal of **9d**, this distance is only 3.054(2) Å, i.e. 0.35 Å less than the sum of the van der Waals radii (the O-6···ring centroid distance is nominally 3.052 Å, i.e., indistinguishable from the C5···C5′ distance).

**Figure 3:**
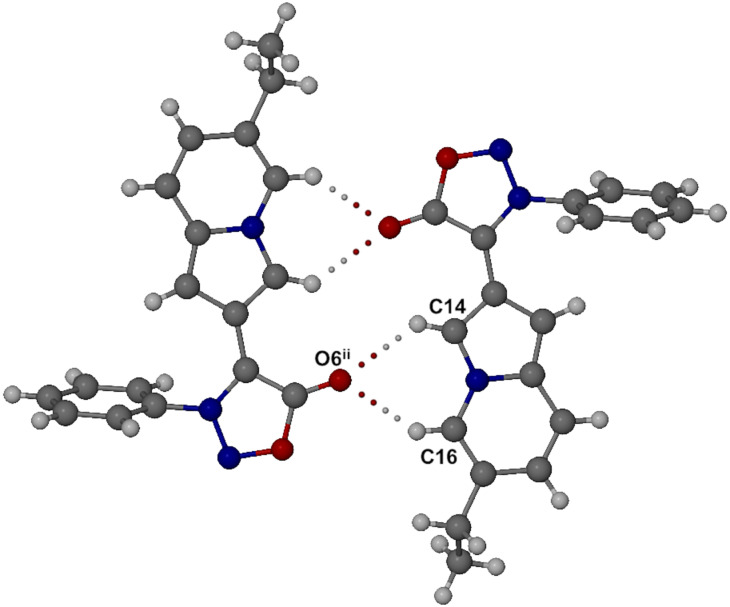
Centrosymmetric C–H···O hydrogen bonded dimeric motif in the crystal of **9d**.

The molecules of **9d** associate into centrosymmetric dimers via two identical pairs of intermolecular bifurcated C–H···O hydrogen bonds (Figure 3), namely C14–H14···O6^ii^ and C16–H16···O6^ii^ (ii = *−x, −y, −z*) with C···O distances of 3.271(2) and 3.216(2) Å, respectively.

In the ^1^H NMR spectra the chemical shifts for the indolizine protons were assigned on the basis of HH and HC correlations and by comparison with NMR data for the parent indolizine. The chemical shifts of hydrogen atoms of compound **9a** substituted at position 2 with a sydnone have different values for H-1 (Δ = 0.6 ppm) and H-3 (Δ = 0.6 ppm) when compared to unsubstituted indolizine whereas the variations in chemical shift data for the other protons are less than 0.2 ppm. It should be mentioned that in the case of compounds **9a**,**c**,**d** the signals for protons H-3 and H-5 overlap with those of the phenyl group protons. The ^13^C NMR spectra of sydnone-indolizines show a good agreement with the parent indolizine **2** and its derivatives. Also noteworthy are the values of the chemical shifts for C-4 (δ = 106.9–107.9 ppm) and C-5 (CO group, δ = 166.9–167.9 ppm) from the sydnone ring. The IR spectra of biheteroaryls **9** show the characteristic absorbance bands of the endocyclic CO group of the sydnone ring in the range 1724–1765 cm^−1^.

### Synthesis of sydnone-indolizines by 1,3-dipolar cycloaddition reaction

As indolizines exhibit more than 30 different types of biological activity and they are highly fluorescent compounds the synthesis of new compounds with an indolizine framework is of great interest. The Chichibabin method and 1,3-dipolar cycloaddition reactions of pyridinium *N*-ylides with acetylenic dipolarophiles are two of the most versatile syntheses for the preparation of indolizines. Both methods use as starting materials pyridinium salts whose preparation is simple and in many cases commercially available pyridines and halogenated compounds are employed.

**Scheme 2:**
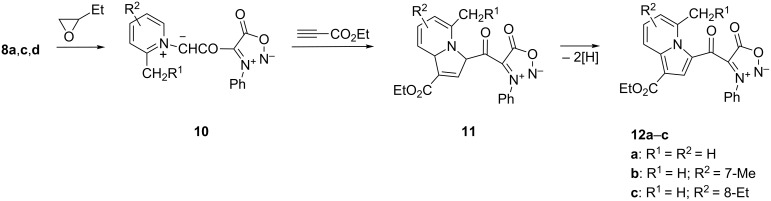
The synthesis and mechanism of formation of sydnone-indolizines **12**.

Therefore, the pyridinium bromides **8** used in the synthesis of bihetaryls **9** can also serve as starting materials for the generation of sydnone-pyridinium *N*-ylides **10** (Scheme 2) in the presence of acetylenic dipolarophiles, to form sydnone-indolizine hybrids **12**. In the new compounds **12** (Scheme 2) the indolizine moiety is connected by a keto group to C-4 of the sydnone. The reaction was achieved in a one-pot procedure by refluxing in 1,2-epoxybutane the bromides **8** with ethyl propiolate as acetylenic dipolarophile. The formation of cycloadducts **12** implies in the first step the generation of pyridinium *N*-ylides under the action of 1,2-epoxybutane. The mechanism for the transformation of *N*-heteroaromatic salts into the corresponding *N*-ylides with epoxides was reported recently [5,17–18]. The next step represents a 1,3-dipolar cycloaddition of the bis(1,3-dipoles) **10** with formation of dihydro-indolizines **11** which, under the reaction conditions, are dehydrogenated to the final products **12a**–**c** with yields in the range of 41–52%.

Bearing in mind that the formation of indolizines through Chichibabin synthesis and 1,3-dipolar cycloaddition reaction requires in both cases the formation of an intermediate pyridinium *N*-ylide, it is expected that in these cycloadditions a mixture of sydnone-indolizine **9** and cycloadduct **12** might result. In order to verify this hypothesis the crude products resulting from the cycloaddition reactions were carefully investigated by NMR spectroscopy. No trace of product **9** that might have resulted from Chichibabin cyclization was observed. This result indicates that the reaction rate of pyridinium *N*-ylides **10** with the acetylenic dipolarophile is fast in comparison with an intramolecular cyclization to form compounds **9**.

**Figure 4:**
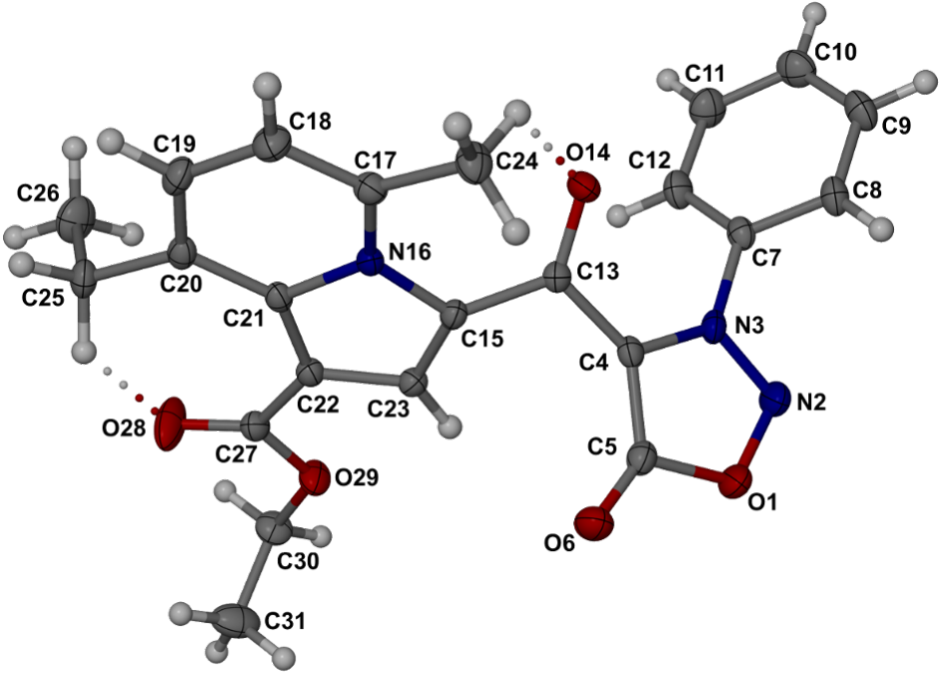
Molecular structure of **12c** with atoms represented as thermal ellipsoids at the 50% probability level. Dotted lines indicate intramolecular hydrogen bonds. Bond distances (Å) in the sydnone moiety are: O1–N2 1.370(2), N2–N3 1.307(2),N3–C4 1.361(2), C4–C5 1.427(2), C5–O1 1.424(2), and C5–O6 1.202(2).

The structures of the sydnone-indolizines **12** were assigned by NMR spectroscopy, IR and elemental analysis. As representative compound, **12c** was selected for X-ray structural elucidation. Figure 4 shows the molecular structure, whose conformation is partially stabilized by two significant intramolecular hydrogen bonds. These are found between C24–H24A···O14 and C25–H25B···O28, with remarkably short C···O distances of 2.787(2) and 2.912(2) Å, respectively. Torsion angles that define the relative orientations of the indolizine moiety, the sydnone ring and the linking keto group include C23–C15–C13–C4 −38.7(2)°, C23–C15–C13–O14 138.0(1)° and C15–C13–C4–C5 −37.3(2)°. The relative orientations of the phenyl and sydnone rings are defined by the torsion angle C4–N3–C7–C12 −55.7(2)°.

**Figure 5:**
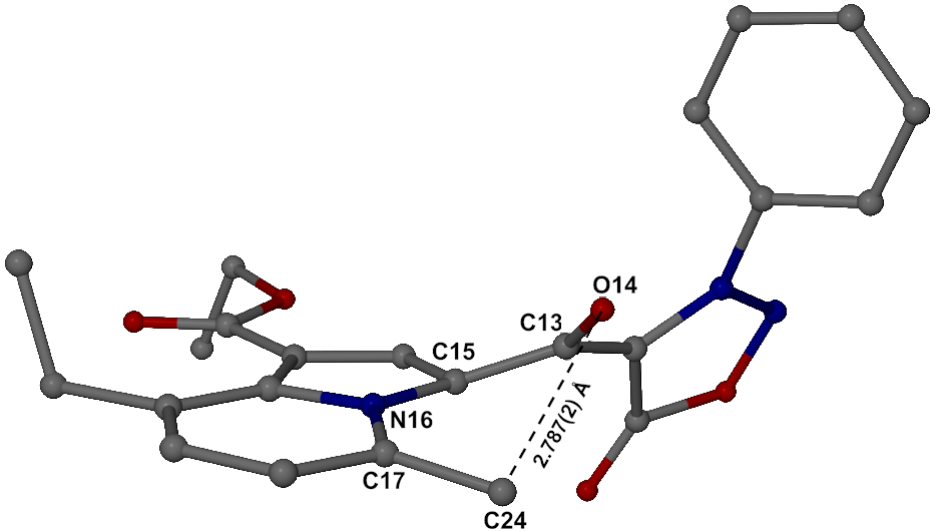
Intramolecular distortion in **12c**.

There is evidence of significant steric hindrance in the ‘bay region’ of the indolizine moiety of **12c**, with very short non-bonded C···C and C···O distances (Figure 5). In particular, steric repulsion between the methyl carbon C24 and the keto oxygen O14 causes the bond C17–C24 to be rotated by 6.5° below the indolizine plane and the bond C15–C13 to be rotated above the indolizine plane by as much as 20.8°. The net effect is that the chain of atoms C24–C17–N16–C15–C13–O14 resembles a spiral ramp.

The introduction of the linking keto group in **12c** also results in small but statistically significant changes in the interatomic distances in the sydnone ring relative to compound **9d**. The most pronounced effect is observed for the distance C5–O1, which is 0.022 Å longer in **12c**, i.e., a difference of ~8σ (σ = combined standard deviation). Furthermore, the sydnone-ring environment in the crystal of **12c** differs significantly from that in **9d**. The dipolar alignment of the sydnone moieties observed in **9d** does not occur in **12c**. The exocyclic atom O6 engages as an acceptor in an intermolecular C–H···O hydrogen bond. Further stabilizing interactions in the crystal of **12c** include two additional intermolecular C–H···O bonds and an offset π-stacking interaction between the indolizine ring and its inversion-related counterpart, with the shortest centroid···centroid distance 3.718(1) Å.

The ^1^H NMR spectra of compounds **12** confirmed the indolizine structure of the cycloadducts. The representative signals for the protons H-2 appear at 8.16–8.18 ppm as strongly deshielded singlets. The protons from the pyridine moiety were assigned unequivocally and could be distinguished from the protons of the sydnone-attached phenyl ring. The ^13^C NMR data are in agreement with the proposed structures for the hybrid heterocycles **12**. The signals for the endocyclic and exocyclic CO groups of the sydnone moiety are strongly deshielded and the chemical shifts are very close, appearing in the range of 165.5–166.1 ppm, whereas the carbonyls of the ester groups are shielded by 2–3 ppm. Both, C-1 from the indolizine and C-4 from the sydnone rings are shielded and resonate in the range of 106.8–106.9 ppm. In the two series of indolizines **9** and **12** the chemical shifts for the carbon atoms of the indolizine moiety are strongly dependent on the nature of the substituents. Thus, the differences between the chemical shifts for the corresponding carbon atoms of the two indolizine series are generally higher than 5 ppm (except for C-3 and C-8). In the ATR-IR spectra the main characteristic absorption frequencies are those for the three carbonyl groups.

## Conclusion

The indolizination of sydnones at C-4 was achieved by the simple Chichibabin indolizine synthesis involving the formation of pyridinium salts from commercially available pyridines and 4-bromoacetyl-3-phenylsydnone, followed by cyclization of the pyridinium bromides in the presence of a base.

Depending on the accessibility of the compounds of type Het-COCH_2_Hal the indolizination of aromatic heterocycles by the Chichibabin synthesis could be extended to link two heteroarenes. This method overcomes the difficulties encountered in obtaining biheteroaryls through palladium-catalyzed cross-coupling reactions.

In addition, the pyridinium salts used in the synthesis of sydnone-indolizine hybrids could be transformed into sydnone-indolizines connected via a keto group. The transformation was achieved by a one-pot procedure implying the in situ generation of sydnone-pyridinium *N*-ylides followed by 1,3-dipolar cycloaddition reaction with an acetylenic dipolarophile.

The structures of the new hybrid heterocyclic sydnone-indolizines were assigned by elemental analysis, NMR and IR spectroscopy and X-ray analyses of representative compounds. The latter technique revealed the dipolar alignment of the sydnone moieties in one case and a significant intramolecular distortion in the other.

## Supporting Information

Experimental details of synthetic procedures and experimental details of X-ray structure analyses. Crystallographic information files (CIF) for compound **9d** and **12c**, listing crystal data, experimental and refinement details, and molecular parameters (bond lengths, bond angles and torsion angles) are provided.

File 1 Experimental.

File 2 Crystallographic information file (CIF) for compound **9d**.

File 3 Crystallographic information file (CIF) for compound **12c**.
