# Supplementary material for: Sydnone C-4 heteroarylation with an indolizine ring via Chichibabin indolizine synthesis
Source: Beilstein J Org Chem. 2016 Nov 23;12:2503–10. doi: 10.3762/bjoc.12.245 (PMC5238577; doi:10.3762/bjoc.12.245)
Supplement: File 1 — Experimental. [file Beilstein_J_Org_Chem-12-2503-s001.pdf]

Supporting Information

for

Sydnone C-4 heteroarylation with an indolizine ring via Chichibabin indolizine synthesis

Florin Albota[1], Mino R. Caira[2]*, Constantin Draghici[1], Florea Dumitrascu[1]*, Denisa E. Dumitrescu[3]

Address: [1]Center of Organic Chemistry ‘‘C. D. Nenitzescu’’, Roumanian Academy, Spl Independentei 202B, 060023 Bucharest, Romania, [2]Department of Chemistry, University of Cape Town, Rondebosch 7701, South Africa and [3]Faculty of Pharmacy, University Ovidius, Aleea Universitatii 1, Constantza, Romania

Email: Dr. Florea Dumitrascu - fdumitra@yahoo.com; Mino R. Caira - Mino.Caira@uct.ac.za

*Corresponding author

Experimental

General

Melting points were determined on a Böetius hot plate microscope. Elemental analysis was carried out on a COSTECH Instruments EAS32 apparatus. The IR spectra were recorded on a FTIR Bruker Vertex 70. The NMR spectra were recorded on a Varian Gemini 300 BB instrument, operating at 300 MHz for ^{1}H NMR and 75 MHz for ^{13}C NMR. Supplementary evidence was given by HETCOR and COSY experiments. X-ray structural elucidation of representative compounds **9d** and **12c** was also achieved.

General procedure for the synthesis of pyridinium bromides 8a–d

Pyridines **6a–d** (8 mmol) and 4-bromoacetyl-3-phenylsydnone **7** (2 g, 7 mmol) were dissolved in 25 mL acetone and the solution was heated under reflux with stirring for 8 h. After cooling of the reaction mixture the precipitate was filtered and washed with acetone on the filter. The crude product was used in the next step without further purification.

2-Methyl-1-[2-(3-phenylsydnon-4-yl)-2-oxoethyl]pyridinium bromide (8a). The compound was purified by crystallization from ethanol. Colorless crystals, mp 156-8 °C. Yield 81 %. IR (ATR): 1771 cm^{-1} ($\nu_{C=O}$ endocyclic), 1691 cm^{-1} ($\nu_{C=O}$ exocyclic). ^{1}H-NMR (300 MHz, DMSO-d_6) δ: 2.69 (s, 3H, Me), 6.05 (s, 2H, CH_2), 7.62-7.78 (m, 5H, Ph), 7.99-8.04 (m, 1H, H-5), 8.08-8.10 (m, 1H, H-3), 8.52-8.57 (m, 1H, H-4), 8.90-8.93 (m, 1H, H-6); ^{13}C-NMR (75 MHz, DMSO-d_6) δ: 19.8 (Me), 63.4 (CH_2CO), 106.2 (C-4, Syd), 125.2, 129.5, 132.6 (5C, Ph), 134.3 (C-1, Ph), 125.4, 129.5, 146.5, 146.8 (C-3, C-4, C-5, C-6), 156.4 (C-2), 165.7 (CO-

endocyclic), 176.4 (CO-exocyclic). Anal. Calcd for $C_{16}H_{14}BrN_3O_3$; C, 51.08; H, 3.75; N, 11.17; found C, 51.36; H, 3.49; N, 11.34.

2-Ethyl-1-[2-(3-phenylsydnon-4-yl)-2-oxoethyl]pyridinium bromide (8b). The compound was purified by crystallization from ethanol. Colorless crystals, mp 205-7 °C. Yield 78 %. IR (ATR): 1763 cm^{-1} ($\nu_{C=O}$ endocyclic), 1683 cm^{-1} ($\nu_{C=O}$ exocyclic); ^{1}H-NMR (300 MHz, DMSO-d_6) δ: 1.24 (t, 3H, *J*=7.4 Hz, $MeCH_2$), 2.96 (q, 2H, *J*=7.4 Hz, CH_2Me), 6.06 (s, 2H, CH_2), 7.61-7.77 (m, 5H, Ph), 7.98-8.03 (m, 1H, H-5), 8.06-8.09 (m, 1H, H-3), 8.56-8.60 (m, 1H, H-4), 8.91-8.93 (m, 1H, H-6); ^{13}C-NMR (75 MHz, DMSO-d_6) δ: 11.9 (Me, Et), 25.3 (CH_2, Et), 63.0 (CH_2CO), 106.3 (C-4, Syd), 125.3, 129.6, 132.7 (6C, 5C-Ph, C-5), 134.3 (C-1, Ph), 127.6, 146.7, 147.1 (C-3, C-4, C-6), 160.4 (C-2), 165.8 (CO-endocyclic), 176.7 (CO-exocyclic). Anal. Calcd for $C_{17}H_{16}BrN_3O_3$; C, 52.32; H, 4.13; N, 10.77; found 52.60; H, 4.42; N, 11.10.

2,4-Dimethyl-1-[2-(3-phenylsydnon-4-yl)-2-oxoethyl]pyridinium bromide (8c). The compound was purified by crystallization from ethanol. Colorless crystals with mp 155-8 °C. Yield 83 %. IR (ATR): 1772 cm^{-1} ($\nu_{C=O}$ endocyclic), 1685 cm^{-1} ($\nu_{C=O}$ exocyclic); ^{1}H-NMR (300 MHz, DMSO-d_6) δ: 2.54, 2.61 (2s, 6H, 2Me), 5.96 (s, 2H, CH_2), 7.61-7.77 (m, 5H, Ph), 7.82 (dd, 1H, *J*=6.6, 1.9 Hz, H-5), 7.91 (d, 1H, *J*=1.9 Hz, H-3), 8.72 (d, 1H, *J*=6.6 Hz, H-6); ^{13}C-NMR (75 MHz, DMSO-d_6) δ: 19.6, 21.4 (Me), 62.7 (CH_2CO), 106.3 (C-4, Syd), 125.3, 129.6, 132.7 (5C, Ph), 134.4 (C-1, Ph), 126.0, 129.7, 145.8 (C-3, C-5, C-6), 155.1, 159.9 (C-2, C-4), 165.8 (CO-endocyclic), 176.8 (CO-exocyclic). Anal. Calcd for $C_{17}H_{16}BrN_3O_3$; C, 52.32; H, 4.13; N, 10.77; found C, 52.51; H, 4.39; N, 10.98.

5-Ethyl-2-methyl-1-[2-(3-phenylsydnon-4-yl)-2-oxoethyl]pyridinium bromide (8d). The compound was purified by crystallization from ethanol. Colorless crystals with mp 221-223 °C. Yield 80 %. IR, (ATR): 1779 cm^{-1} ($\nu_{C=O}$ endocyclic), 1689 cm^{-1} ($\nu_{C=O}$ exocyclic); ^{1}H-NMR (300 MHz, DMSO-d_6) δ: 1.20 (t, 3H, *J*=7.4 Hz, $MeCH_2$), 2.63 (s, 3H, Me), 2.74 (q, 2H, *J*=7.4 Hz, CH_2Me), 6.00 (s, 2H, CH_2), 7.61-7.71, 7.75-7.78 (2m, 5H, Ph), 7.99 (d, 1H, *J*=8.2, H-3), 8.44 (dd, 1H, *J*=8.2, 1.9 Hz, H-4), 8.85 (dd, 1H, *J*=1.9 Hz, H-6); ^{13}C-NMR (75 MHz, DMSO-d_6) δ: 14.1 (Me, $MeCH_2$), 19.4 (Me), 24.6 (CH2, $MeCH_2$), 63.4 (CH_2CO), 106.2 (C-4, Syd), 125.3, 129.6, 132.7 (5C, Ph), 134.3 (C-1, Ph), 129.1, 145.4, 146.1 (C-3, C-4, C-6), 141.3, 153.9 (C-2, C-5), 165.7 (CO-endocyclic), 176.4 (CO-exocyclic). Anal. Calcd for $C_{18}H_{18}BrN_3O_3$; C, 53.48; H, 4.49; N, 10.39; found C, 53.80; H, 4.84; N, 10.67.

General procedure for Chichibabin synthesis of indolizines 9a–d

The crude pyridinium bromides **8** (3 mmol) were dissolved with stirring in 40 mL of hot water containing two grams of sodium bicarbonate. Afterwards the reaction mixture was heated under stirring at 80–90 °C for 1 h. After cooling, the indolizines **9** were isolated from the reaction mixture by extraction with methylene chloride followed by purification by column chromatography using methylene chloride as eluent.

2-(3-Phenylsydnon-4-yl)indolizine (9a). The compound was purified by crystallization from methanol. Light brown crystals, mp 152-55 °C; Yield 51 %. IR (ATR): 1734 cm^{-1} ($\nu_{C=O}$ endocyclic); ^{1}H-NMR (300 MHz, $CDCl_3$) δ: 5.76-5.77 (m,

1H, H-1), 6.42-6.47 (m, 1H, H-6), 6.59-6.65 (m, 1H, H-7), 7.10-7.14 (m, 1H, H-8), 7.59-7.70 (m, 5H, Ph), 7.73-7.76 (m, 1H, H-3), 7.78-7.81 (m, 1H, H-5); ^{13}C-NMR (75 MHz, $CDCl_3$) δ: 95.6 (C-1), 106.9 (C4-Syd), 111.2, 111.4 (2C, C-3,C-6), 112.3 (C-2), 118.4, 118.9 (2C, C-7, C-8); 125.2 (C-5); 125.6, 130.2, 132.4, 134.7 (6C, Ph); 132.9 (C-8a); 166.9 (CO-endocyclic). Anal. Calcd for $C_{16}H_{11}N_3O_2$; C, 69.31; H, 4.00; N, 15.15; found 69.62; H, 4.29; N, 15.42.

1-Methyl-2-(3-phenylsydnon-4-yl)indolizine (9b). The compound was purified by crystallization from methanol. Colorless crystals, mp 154-6 °C; yield 69 %. IR (ATR): 1735 cm^{-1} ($\nu_{C=O}$ endocyclic); ^{1}H-NMR (300 MHz, $CDCl_3$) δ: 1.88 (s, 3H, Me), 6.40-6.45 (m, 1H, H-6), 6.56-6.62 (m, 1H, H-7), 7.18-7.21 (m, 1H, H-8), 7.28-7.29 (m, 1H, H-3), 7.47-7.60 (m, 5H, Ph), 7.72-7.74 (m,1H, H-5); ^{13}C-NMR (75 MHz, $CDCl_3$) δ: 9.3 (Me), 104.6 (C-1), 107.9 (C4-Syd), 110.8 (C-2), 111.2, 111.9 (2C, C-3. C-6), 116.5, 117.7 (2C, C-7, C-8), 124.2, 129.9, 131.7, 135.1 (6C-Ph), 125.0 (C-5), 130.8 (C-8a), 167.9 (CO-endocyclic). Anal. Calcd for $C_{17}H_{13}N_3O_2$; C, 70.09; H, 4.50; N, 14.42; found C, 70.41; H, 4.81; N, 14.71.

7-Methyl-2-(3-phenylsydnon-4-yl)indolizine (9c). The compound was purified by crystallization from ethanol. Colorless crystals, mp 114-6 °C; yield 61 %. IR (ATR): 1724 cm^{-1} ($\nu_{C=O}$ endocyclic); ^{1}H-NMR (300 MHz, $CDCl_3$) δ: 2.19 (s, 3H, Me), 5.59-5.61 (m, 1H, H-1),6.27-6.30 (m, 1H, H-6), 6.86-6.88 (m, 1H, H-8), 7.58-7.77 (m, 7H, H-3, H-5, Ph); ^{13}C-NMR (75 MHz, $CDCl_3$) δ: 21.1 (Me), 94.0 (C-1), 106.9 (C4-Syd), 110.6 (C-2), 112.2 (C-6), 114.2 (C-3); 116.8 (C-8); 124.7 (C-5); 125.7, 130.1, 132.3, 134.7 (6C, Ph); 128.7 (C-7); 133.3 (C-8a), 166.9 (CO-

endocyclic). Anal. Calcd for $C_{17}H_{13}N_3O_2$; C, 70.09; H, 4.50; N, 14.42; found C, 70.41; H, 4.77; N, 14.64.

6-Ethyl-2-(3-phenylsydnon-4-yl)indolizine (9d). The compound was purified by crystallization from methanol. Light brown crystals, mp 127-9 °C; yield 75 %. IR (ATR): 1765 cm^{-1} ($ν_{C=O}$ endocyclic); ^{1}H-NMR (300 MHz, $CDCl_3$) δ: 1.12 (t, 3H, *J*=7.42, e), 2.42 (q, 2H, *J*=7.42, CH_2), 5.69 (s, 1H, H-1), 6.45-6.49 (m, 1H, H-7), 6.99-7.02 (m, 1H, H-8), 7.52-7.62 (m, 6h, H-3, Ph), 7.66-7.71 (m, 1H, H-5); ^{13}C-NMR (75 MHz, $CDCl_3$) δ: 14.7 (Me), 25.8 (CH_2), 95.3 (C-1), 106.8 (C4-Syd), 110.9, 121.8 (2C, C-3. C-5), 111.7 (C-2), 118.4 (C-8), 120.8 (C-7), 125.6, 130.1, 132.3, 134.7 (6C, Ph), 127.1 (C-6), 132.3 (C-8a), 166.9 (CO-endocyclic). Anal. Calcd for $C_{18}H_{15}N_3O_2$; C, 70.81; H, 4.95; N, 13.76; found C, 71.07; H, 5.21; N, 13.92.

General procedure for synthesis of indolizine cycloadducts 12a–c

Pyridinium bromides **8a**,**c**,**d** (3 mmol) and ethyl propiolate (3.5 mmol) were added to 7 mL 1,2-epoxybutane and the reaction mixture was heated under reflux with stirring for 10 h. The solvent was evaporated under reduced pressure and the residue was triturated with ethanol. The crystalline product was filtered by suction and washed with cold ethanol on the filter. The purification of cycloadducts was achieved by crystallization from an appropriate solvent or by column chromatography on aluminium oxide 90 (Merck, 70–230 mesh) using dichloromethane as eluent.

Ethyl 5-methyl-3-[(3-phenylsydnon-4-yl)-oxomethyl]indolizine-1-carboxylate (12a). The compound was purified by crystallization from a mixture of ethanol/acetonitrile 2:1. Yellow crystals, mp 200-202 °C; yield 41 %. IR (ATR): 1758 cm^{-1} ($\nu_{C=O}$ endocyclic), 1690 cm^{-1} ($\nu_{C=O}$ exocyclic); ^{1}H-NMR (300 MHz, $CDCl_3$) δ: 1.37 (t, 3H, *J*=7.1 Hz, Me), 2.42 (s, 3H, Me), 4.38 (q, 2H, *J*=7.1 Hz, CH_2), 6.82-6.84 (m, 1H, H-6), 7.36-7.41 (m, 1H, H-7), 7.50-7.60 (m, 5H, Ph), 8.17 (s, 1H, H-2), 8.31-8.34 (m, 1H, H-8); ^{13}C-NMR (75 MHz, $CDCl_3$) δ: 14.7 (Me), 23.3 (Me-het), 60.3 (CH_2), 106.8, 107.1 (C-1, C-4-Syd), 117.3 (C-8), 117.6 (C-6), 123.9, 140.5, 143.1 (C-3, C-5, C-8a), 124.2, 129.8, 132.4, 135.2 (6C, Ph), 128.7 (C-7), 130.3 (C-2), 163.8 (COOEt), 165.9 (CO-endocyclic), 166.1 (CO-exocyclic). Anal. Calcd for $C_{21}H_{17}N_3O_5$; C, 64.45; H, 4.38; N, 10.74; found C, 64.37; H, 4.29; N, 10.97.

Ethyl 5,7-dimethyl-3-[(3-phenylsydnon-4-yl)-oxomethyl]indolizine-1-carboxylate (12b). The compound was purified by crystallization from acetonitrile. Yellow crystals, mp 214-6 °C; yield 49 %. IR (ATR): 1768 cm^{-1} ($\nu_{C=O}$ endocyclic), 1686 cm^{-1} ($\nu_{C=O}$ exocyclic); ^{1}H-NMR (300 MHz, $CDCl_3$) δ: 1.42 (t, 3H, *J*=7.2 Hz, $MeCH_2$), 2.44, 2.46 (2s, 6H, 2Me), 7.39 (q, 2H, *J*=7.2 Hz, $MeCH_2$), 6.73 (s, 1H, H-6), 7.50-7.65 (m, 5H, Ph), 8.18 (s, 2H, H-2, H-8); ^{13}C-NMR (75 MHz, $CDCl_3$) δ: 14.6 ($MeCH_2$), 21.4, 22.9 (2Me), 60.2 (CH_2O), 106.0, 107.0 (C-1, C4-Syd), 116.4 (C-8), 120.0 (C-6), 123.5, 139.9, 140.6, 143.6 (C-3, C-5. C-7, C-8a), 124.2, 129.7, 132.3, 135.2 (6C, Ph), 130.8 (C-2); 163.9 (COOEt), 165.6 (CO-endocyclic), 166.0 (CO-exocyclic). Anal. Calcd for $C_{22}H_{19}N_3O_5$; C, 65.18; H, 4.72; N, 10.36; found C, 65.37; H, 4.98; N, 10.64.

Ethyl 8-ethyl-5-methyl-3-[(3-phenylsydnon-4-yl)-oxomethyl]-indolizine-1-carboxylate (12c). The compound was purified by crystallization from acetonitrile. Orange crystals, mp 207-9 °C; yield 52 %. IR (ATR): 1751 cm^{-1} ($\nu_{C=O}$ endocyclic), 1681 cm^{-1} ($\nu_{C=O}$ exocyclic); ^{1}H-NMR (300 MHz, $CDCl_3$) δ: 1.16 (t, 3H, *J*=7.4 Hz, $MeCH_2$-Ind), 1.35 (t, 3H, *J*=7.1 Hz, $MeCH_2$-O), 2.35 (s, 3H, Me-Ind), 3.23 (q, 2H, *J*=7.4, CH_2-Ind), 4.30 (q, 2H, *J*=7.1, CH_2-O), 6.78, 7.21 (2d, 2H, *J*=7.4 Hz, H-6, H-7), 7.49-7.57 (m, 5H, Ph), 8.16 (s, 1H, H-2); ^{13}C-NMR (75 MHz, $CDCl_3$) δ: 14.6 ($MeCH_2$-Ind), 15.3 ($MeCH_2$-O), 23.3 (Me-Ind), 26.9 (CH_2-Ind), 60.7 (CH_2-O), 106.8 (C-1), 109.2 (C-4-Syd), 117.9 (C-6), 123.4, 133.9, 138.2, 141.3 (C-3, C-5, C-8, C-8a), 124.2, 129.7, 132.1, 135.3 (6C, Ph), 128.8 (C-7), 132.3 (C-2), 163.9 (COOEt), 165.5 (CO-endocyclic), 166.0 (CO-exocyclic). Anal. Calcd for $C_{23}H_{21}N_3O_5$; C, 65.86; H, 5.05; N, 10.02; found C, 66.18; H, 5.41; N, 10.33.

X-ray structural analysis

Intensity data for crystals of **9d** and **12c** were collected on a Nonius Kappa CCD diffractometer and a Bruker Apex II diffractometer respectively with MoKα X-rays (λ = 0.71073 Å) with the specimens cooled to 173(2) K in a nitrogen stream. Data reduction programs employed are listed in the CIF files (Supporting Information File 2 (**9d**) and Supporting Information File 3 (**12c**). For structure solution by direct methods and full-matrix least-squares refinements, the programs in the SHELX suite were employed [1]. Following isotropic refinement of non-hydrogen atoms, anisotropic thermal displacement parameters were introduced. All H

atoms were located in difference electron density maps and were included in a riding model with U_{iso} values fixed at 1.2–1.5 times those of their parent atoms.

Crystal data for compound 9d. $C_{18}H_{15}N_3O_3$, MW = 305.33, monoclinic, *a* = 11.7917(5) Å, *b* = 7.5448(3) Å, *c* = 17.6610(8) Å, β = 101.754(2)°, *V* = 1538.28(11) Å^3, *T* = 173(2) K, space group $P2_1$/c, *Z* = 4, D_c = 1.318 g cm^{-3}, μ (MoKα) = 0.088 mm^{-1}, θ range for data collection = 1.00-27.48, -15 ≤ h ≤ 15, -9 ≤ k ≤ 9, -22 ≤ l ≤ 22, 6723 reflections collected, 3492 unique (R_{int} = 0.0352), 2561 with *I* >2σ(*I*), completeness to θ_{max} = 99.2%, F_{000} = 640, 209 parameters refined, *S* = 1.016, R_1(*I* >2σ(*I*)) = 0.0421, wR_2(all data) = 0.1120, largest diff. peak/hole = 0.252/−0.300 *e* Å^{-3}, CCDC deposition number 1491187.

Crystal data for compound 12c. $C_{23}H_{21}N_3O_5$, MW = 419.43, triclinic, *a* = 8.9685(5) Å, *b* = 10.6487(7) Å, *c* = 11.0785(7) Å, α = 93.480(1)°, β = 95.886(1)°, γ = 109.306(1)°, *V* = 988.21(11) Å^3, *T* = 173(2) K, space group *P(−1)*, *Z* = 2, D_c = 1.410 g cm^{-3}, μ (MoKα) = 0.101 mm-1, θ-range for data collection = 1.86–27.15, −11 ≤ h ≤ 11, −13 ≤ k ≤ 13, −14 ≤ l ≤ 14, 16825 reflections collected, 4376 unique (R_{int} = 0.0407), 3586 with *I* >2σ(*I*), completeness to θ_{max} = 99.7%, F_{000} = 440, 283 parameters refined, *S* = 1.045, R_1(*I* >2σ(*I*)) = 0.0390, wR_2(all data) = 0.1014, largest diff. peak/hole = 0.229/−0.209 *e* Å^{-3}, CCDC deposition number 1491193.

References

1. Sheldrick, G. M. *Acta Crystallogr.* **2008**, *A64*, 112-122.
